# Evaluation of Biocomposite Cements for Bone Defect Repair in Rat Models

**DOI:** 10.3390/life14091097

**Published:** 2024-08-30

**Authors:** Alina Ioana Ardelean, Sorin Marian Mârza, Raluca Marica, Mădălina Florina Dragomir, Alina Oana Rusu-Moldovan, Mărioara Moldovan, Paula Maria Pașca, Liviu Oana

**Affiliations:** 1 Department of Veterinary Surgery, Faculty of Veterinary Medicine, University of Agricultura Sciencies and Veterinary Medicine, 3–5 Manastur Street, 400372 Cluj-Napoca, Romania; alina-ioana.ardelean@usamvcluj.ro (A.I.A.); madalina.dragomir@usamvcluj.ro (M.F.D.); oanaliviu2008@yahoo.com (L.O.); 2 Department of Veterinary Imagistics, Faculty of Veterinary Medicine, University of Agricultura Sciencies and Veterinary Medicine, 3–5 Manastur Street, 400372 Cluj-Napoca, Romania; 3 Department of Veterinary Pathology, Faculty of Veterinary Medicine, University of Agricultura Sciencies and Veterinary Medicine, 3–5 Manastur Street, 400372 Cluj-Napoca, Romania; 4 Department of Surgery III, Institute of Oncology “Prof. Dr. Alexandru Trestioreanu”, 022328 Bucharest, Romania; alinaomoldovan@yahoo.com; 5 Raluca Ripan Institute for Research in Chemistry, Babeș-Bolyai University, 30 Fantanele Street, 400294 Cluj-Napoca, Romania; marioara.moldovan@ubbcluj.ro; 6 Clinics Department, Faculty of Veterinary Medicine, University of Agricultural Science and Veterinary Medicine, 700489 Iasi, Romania; paula.pasca@iuls.ro

**Keywords:** sub-critical bone defect repairment, rats, femur, biomaterial, composite cement scaffolds

## Abstract

Repairing or reconstructing significant bone defects is typically challenging. In the present study, two composite cements were used as scaffolds in a sub-critical femoral defect in rats. A control group and two experimental batches were used to compare the outcomes. This research aimed to investigate the osteogenic potential and toxicological tolerance of the bioproducts through histopathology and computed tomography imaging analysis at 14, 28, 56, and 90 days post-implantation. The biomaterials used in the investigation consisted of a 65% bioactive salinized inorganic filler and a 25% weight organic matrix. The organic part of the biomaterial was composed of Bis-GMA (bisphenol A-glycidyl methacrylate), UDMA (urethane dimethacrylate), HEMA (2-Hydroxyethyl methacrylate), and TEGDMA (triethylene glycol dimethacrylate), while the inorganic filler was composed of silica, barium glass, hydroxyapatite, and fluor aluminosilicate glass. The first findings of this research are encouraging, revealing that there is a slight difference between the groups treated with biomaterials, but it might be an effective approach for managing bone abnormalities. Material C1 exhibited a faster bone defect healing time compared to material C2, where bone fractures occurred in some individuals. It is unclear if the fractures were caused by the presence of the biomaterial C2 or whether additional variables were to blame. By the end of the research, the mice appeared to tolerate the biomaterials without exhibiting any inflammatory or rejection responses.

## 1. Introduction

The body’s bone structure has an extraordinary capability to regenerate and restore itself. The thickness and organization of skeletal trabeculae, morphology, shape, size, and especially the thickness of the cortical area all have a substantial impact on the physical and mechanical properties of the skeleton [1].

Bone abnormalities can arise as a result of trauma, neoplasia, or infection. While autologous grafts are considered the gold standard in managing such abnormalities, restricted accessibility and associated comorbidities limit their widespread use [2,3]. Synthetic skeletal–prosthetic polymers suggest promising results for the restoration of orthopedic defects [4]. In orthopedics, cement scaffolds are commonly utilized to replace skeletal grafts. The mineral structure of the cements is comparable to the composition of bone, which can create a powerful connection with the bone cells [5,6]. Bone cement is utilized in the musculoskeletal domain for filling and restoring abnormal trauma sites. They are frequently employed in different orthopedic and dentistry fixations [7,8]. Despite their exceptional performance, the relatively slow disintegration rate of these compounds limits their broader clinical use. Balancing the decomposition speed of polymers with new bone development is still an issue that needs to be addressed [9].

Because of its superior mechanical qualities, Bis-GMA is a resin commonly used in oral healthcare [10,11,12,13]. The material’s elasticity, longevity, and polymerization shrinkage all increase when a UDMA monomer is used in its stead [14,15,16,17]. Given their important tissue interactions, they encourage angiogenesis and bone formation [18,19,20]. An additional field where cement has been used substantially is orthopedics. PMMA, or polymethyl methacrylate, is a synthetic substance that has no biological activity and is often utilized to secure implants. PMMA is mostly used in hip and knee replacements. Its functionality is based on a mechanical interlock between the prosthesis and the irregularly shaped bone. Bone cement implantation syndrome is frequently linked to this material [21]. Moreover, blood or saline irrigation of the bone can influence PMMA’s mechanical characteristics and impede bone growth. The mechanical characteristics of PMMA are mainly impacted by the addition of hydroxyapatite (HAP), which has been observed to either increase or decrease its strength [22,23]. Despite the implant’s biological compatibility, research has demonstrated that different implants can cause an immediate inflammatory reaction that occasionally results in systemic problems [24]. However, tight control over their structure can successfully limit toxicity and biocompatibility [25].

Based on its biological activity, hydroxyapatite (HAP), a naturally occurring material known as calcium apatite [26], is used in ceramics in medicine. It can exhibit bacteriostatic properties and can stimulate bone regeneration, increasing the survival rate of PMMA bonding. Barium promotes cell adhesion and proliferation as well as the synthesis of angiogenic agents [27].

During new bone formation, certain biomaterials break down within the organism. The byproducts of the decomposition from the material induce little to no tissue reaction, and in other situations, they encourage bone regeneration. The compounds are regularly absorbed or eliminated from cells, and therefore it is essential to pay attention to the organs that process waste [9].

Long bones are developed by endochondral ossification, which appears throughout growth or fracture repair. During this stage, osteogenic cells replace intermediate cartilage, resulting in new bone [28]. Among all bones, the femur tends to be frequently utilized considering its length, causing it to be more suitable for surgical approaches [29].

Unlike the majority of other organs, long bones have the potential to heal automatically with little to no scarring [30].

A ‘critical size defect’ in bone refers to a skeletal injury that will not heal without treatment. By conventional description, it is the smallest structural abnormality that will not fully repair within an animal’s natural life [31,32]. Nonetheless, these essential size differences should be differentiated from abnormalities in which nonunion is induced by a disease-causing mechanism rather than size [33].

Our model evaluates a load-bearing sub-critical bone lesion. Critical or sub-critical size bone defects can arise due to a variety of pathologies, including acute trauma, malignancies, hereditary anomalies, and severe infection [34]. In prior investigations, the systemic and local tissular biocompatibility of the materials was established using in vitro [35] and in vivo cutaneous [36], subcutaneous, and intramuscular testing [37], and the composite cements exhibited osteo-inductive activity without triggering a foreign body reaction.

Various reasons have been suggested in the literature as potential causes of the decreased durability of biomaterials, including debonding, residual air bubbles, cement cracks, and porosity, known as external factors. A number of internal aspects may affect a material’s characteristics: structure, dimensions, form, arrangement of the particles, ratio, and impurities (blood, bone fragments, fluids) [38,39,40]. A detailed description of the cements, including the manufacturing process, has been provided in a previous study [35]. It is well established that the mixing techniques employed significantly influence the quality of the bone cement [40].

The purpose of this study was to evaluate the composite cements in vivo using a certain research method for bone restoration.

Animal models are the foundation of preclinical translational technology advancements. The rats should have as low a morbidity or mortality ratio as possible to yield accurate data [34]. There are various animal models used for bone development, such as mice, rats, rabbits, dogs, pigs, sheep, and goats, but rodent models have been predominant in practically all investigations due to their dimensions, affordability, accessibility, and simplicity of handling [31,41,42]. Rats possess the majority of the main human bones and a similar skeletal structure compared to humans [43]. However, there are specific limitations due to Haversian remodeling [44].

The primary goal of this research was to provide a novel technique for a sub-critical bone defect on a rat’s femoral mid-diaphysis to give suitable clinical relevance. We also assessed the potential of bone to biologically regenerate over four distinct periods with and without external intervention.

## 2. Materials and Methods

### 2.1. Preparation of Bone Biomaterials

The materials were produced at the Raluca Ripan Institute for Research in Chemistry (ICCRR) in Cluj-Napoca, Romania.

**Figure 1:**
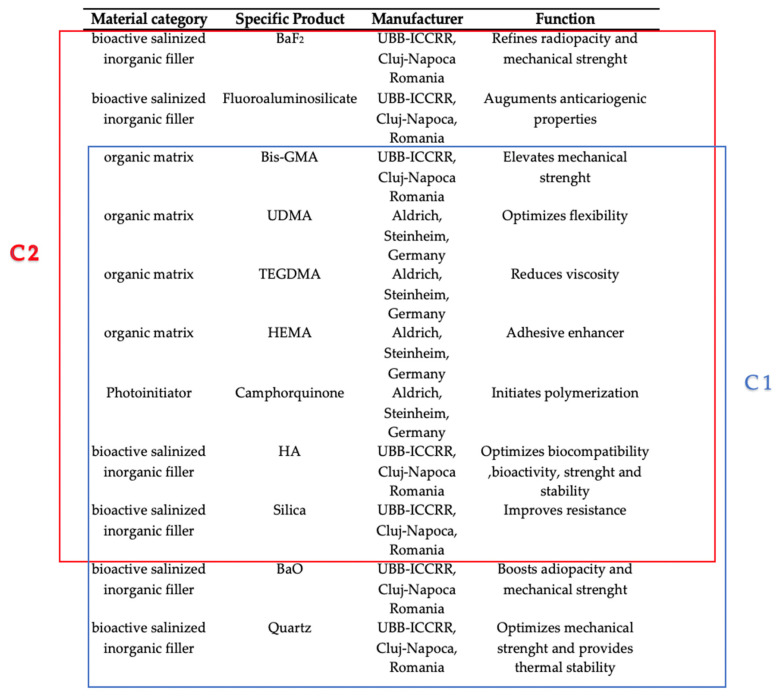
The composition of experimental materials and the functions of the components.

The biomaterials used in this investigation consisted of a 65% bioactive salinized inorganic filler and a 35% weight organic matrix. The organic part of the material was composed of Bis-GMA (ICCRR-UBB, Cluj-Napoca, Romania) ((2,2-bis[p-(2′-hydroxy-3′-metacryloxypropoxy)phenyl]-propane), UDMA (urethane dimethacrylate), HEMA (hydroxyethyl methacrylate), and TEGDMA (triethylene glycol dimethacrylate) (Sigma Aldrich in Darmstadt, Germany) while the inorganic filler was composed of silica, barium glass, hydroxyapatite, and fluor aluminosilicate glass (synthesized to ICCRR-UBB, Cluj-Napoca, Romania). The research led to the development of bioproducts C1 and C2 by distributing particles in the organic phase. The photopolymerization phase was initiated with a camphorquinone photoinitiator (CQ) (0.5% relative to the liquid mixture)/amine (1%) as the initiator/activator using an O-Star Curing Light lamp (Guilin Woodpecker Medical Instrument, Co., Ltd., Guilin, China) for 20 s. The composition of both products is illustrated in Figure 1.

The microstructural characteristics and elemental composition of the cements were thoroughly examined and documented in a previous investigation conducted at our research facility. Moreover, early in vitro research was conducted before creating in vivo experiments. The research of Ardelean et al. offers all the data needed [35].

**Figure 2:**
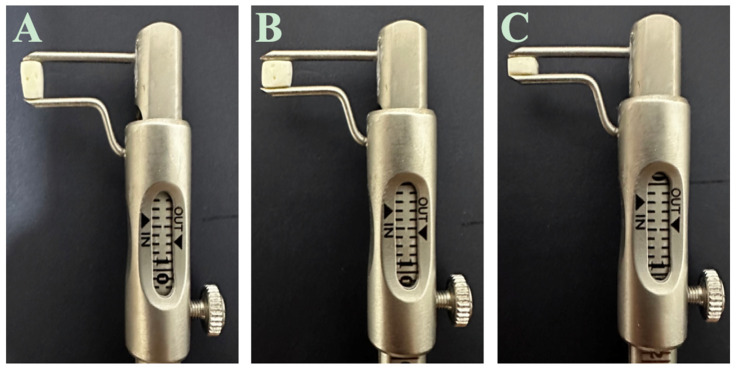
The representation of the biomaterial C1 in height (**A**), width (**B**), and length (**C**).

Before insertion, the biomaterials were prepared to match the defect in a cuboidal shape which measured 4.00 mm in length, 2.00 mm in height, and 3 mm in width (Figure 2). The cements were sterilized on the day of surgery to prevent contamination.

The biomaterials were autoclaved at 105 °C for 10 min using a Trade Raypa Steam Sterilizer (R. Espinar, S.L., Spania, AE-75 Dry) to decontaminate them before the implantation.

### 2.2. Ethics Statement

This research study was carried out at the Faculty of Veterinary Medicine’s Establishment for Breeding and Use of Laboratory Animals in Cluj-Napoca, Romania, after the specimens had been obtained from the Experimental Medicine Center at the University of Medicine and Pharmacy Iuliu Hatieganu.

According to standards, the individuals used in the investigation received regular care, with consistent feeding intervals and closely monitored living conditions, such as a temperature of 23 °C, humidity cycles of 55%, and light/dark cycles of 12 h.

This study received approval from the Bioethics Committee of the University of Agricultural Sciences and Veterinary Medicine Cluj-Napoca no. 352/12.12.2022 and authorized by the Sanitary–Veterinary and Food Safety Department, Cluj-Napoca, through the Project Authorization no. 374/04.07.2023.

### 2.3. Blood Tests

Hematology and biochemistry investigations were conducted to confirm the rats’ health condition. Blood was collected after the rats had been sedated, in specific blood-collecting tubes. A capillary tube was implanted at an angle of 30 degrees in the medial canthus of the eye [45]. The results were compared to specific literature [46,47].

#### 2.3.1. Hematology Blood Test

Blood was processed with Abaxis VetScan HM5 hematology analyzer (Abaxis Inc., Union City, CA, USA). The full blood count was performed to measure the following: WBCs: White Blood Cells, LYMs: Lymphocytes, MONs: Monocytes, NEUs: Neutrophils, RBCs: Red Blood Cells, HCT: Hematocrit, HGB: Hemoglobin, MCV: Mean Cell Volume, MCH: Mean Corpuscular Hemoglobin, MCHC: Mean Corpuscular Hemoglobin Concentration, and PLT: Platelet count.

#### 2.3.2. Biochemistry Blood Test

The blood was processed with an Automatic Veterinary Chemistry Analyzer Element RC (Scil Animal Care Company, Alfort, France). Several parameters were evaluated, such as ALB: Albumin, TP: Total Protein, TB: Total Bilirubin, ALT: Alanine Aminotransferase, ALP: Alkaline Phosphatase, CREA: Creatinine, UREA: Blood Urea Nitrogen, GLU: Glucose, CA: Calcium, PHOS: Phosphorus, K: Potassium, and NA: Sodium.

### 2.4. Animal Care and Use

For the animal resource in this investigation, we employed 24 adult female rats, divided into three groups, as follows: a control group, comprising 8 rats with no product (C0); group 1, composed of 8 rats with Cement 1 (C1); and group 2, consisting of 8 rats with Cement 2 (C2). The research rodent species were from the Muridae family, specifically the Wistar-Lewis line, around 350 g in weight and 10 weeks old. This variety was chosen because of its special attribute [48].

On days 0, 14, 28, 56, and 90, CT scans were performed. Blood samples were obtained on the first and last days of the research. Tissue samples were obtained for histological examination on days 14, 28, 56, and 90.

Two individuals per group were painlessly sacrificed through cervical dislocation while under general anesthesia, in conformity with international protocols, on each sacrificial day; the sacrificial days were days 14, 28, 56, and 90.

This study was carried out at the Faculty of Veterinary Medicine’s Establishment for Breeding and Use of Laboratory Animals in Cluj-Napoca, Romania, after the specimens had been obtained from the Experimental Medicine Center at the University of Medicine and Pharmacy Iuliu Hatieganu.

### 2.5. Surgical Procedure

#### 2.5.1. Anesthesiologic Protocol and Pain Control

To make the surgical process less unpleasant, we used an induction cage for the rodents. The anesthetic agent employed was isoflurane (Isothesia 250 mL, Omegavet, Bucuresti, Romania). The specimens remained in the box until the point at which they were unconscious, after which the intraperitoneal medication was administered. Rodents received anesthesia based on their body mass from a combination of ketamine (50 mg/kg Narkamon Bio, Bioveta, Ivanovice na Hané, Czech Republic) and medetomidine (0.25 mg/kg Domitor, Biotur, Teleorman, Romania), injected intraperitoneally.

The rodents’ eyes were protected against dryness using Corneregel eye gel (Bausch & Lomb, Rochester, NY, USA).

Following surgery, the rats received supplementary oxygen. Postoperatively, for analgesia we used Buprenorphine (1 mg/kg Bupaq, Biotur, Teleorman, Romania) subcutaneously in the first, second, and third days, one dose per 24 h.

#### 2.5.2. Surgical Protocol

The animals were positioned in latero-lateral recumbency. From the sacral vertebrae to the tail and tarsal area, the right leg was clipped and aseptically prepared with diluted 4% Chlorhexidine and 70% Sanitary Alcohol. A 25 mm longitudinal incision was made with a #10 scalpel blade on the lateral side of the femur while holding it at full extension. The subcutaneous tissue was dilacerated with blunt dissection to expose the fascia of the Vastus Lateralis, Gluteal, and Biceps Femoris muscles. The dissection continued until the femur was exposed. Miniature Senn–Miller retractors were used to retract the muscular tissue, allowing for better visualization. Using an #11 scalpel blade, the periosteum was gently cut and lateralized out of the bone with a moisturized sterile Q-tip. The femur mid-diaphysis was then visible and prepared to create the bone defect.

Due to the modest size of the diaphysis, the bone defects were made using a neurosurgical 2 mm drill with a slow rotation speed (1500 rpm). During the defect creation, special care was given to the nutrient foramen to preserve it. The bone was cooled with a NaCl 0.9% (Chloride Sodium 0.9%, Braun, Ilfov, Romania) solution while drilling, using a 10 mL syringe and a 21 G needle to avoid overheating and cell apoptosis. Additionally, bone fragments and bone powder were carefully removed with this lavage to limit potential damage.

**Figure 3:**
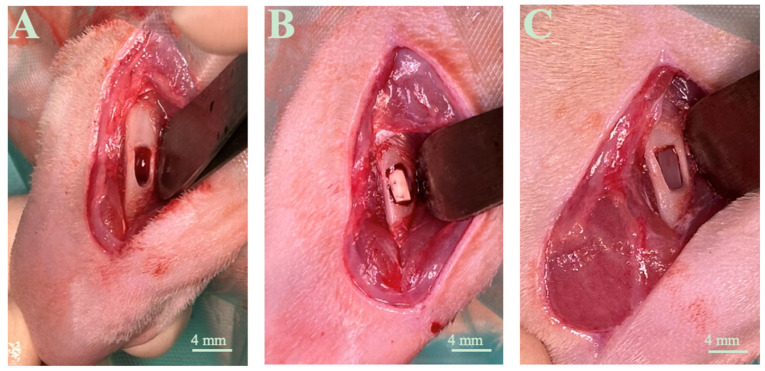
The bone defect was performed on the middle segment of the rats’ femoral diaphysis of the right limb. Lateral exposure of the defect without (**A**) and with biomaterial C1 (**B**) and biomaterial C2 (**C**) on day 0.

The defect’s size was measured using a mechanical millimetric caliper. A cortical rounded-rectangular window 4.1 mm in length, 2.1 mm in height, and 3.1 mm in width (Figure 3A) was produced in the midsection of the diaphysis. The first group was the control group (C0). Their wound had the opportunity to heal naturally without any exterior interference. The second and third groups were provided with the C1 and C2 polymerized biomaterials (Figure 3B,C).

The defect and surrounding tissue were lavaged with NaCl 0.9% solution before being closed in layers: muscle fascia with a simple interrupted pattern and subcutaneous tissue with a few buried knots using a 5.0 atraumatic absorbable polydioxanone monofilament (PDO) (BioSintex, Snagov, Ilfov, Romania). The skin was closed using 4.0 nonabsorbable polyamide traumatic monofilament Nylon (BioSintex, BioNil Mono, Snagov, Ilfov, Romania) wire in a simple interrupted pattern.

### 2.6. Imaging

Starting on day one, all rats were scanned to observe the progression of the lesion. Aside from the progress, we also considered the possibility of fracture occurrence due to the destabilization of the femur.

Helical CT scanning of the right hind limb was obtained using a Siemens CT Somatom Scope machine with 16 channels. The scan was performed with the patient in sterno-abdominal recumbency. The patient was fully sedated using inhalation anesthetics (Isothesia, Baia-Mare, Romania).

Body images were obtained in the axial plane using a lower extremities protocol scan with a 512 × 512 matrix, narrow windows (WW: 120, WL: 40), 3 mm slice thickness, and a pitch of 3 mm, at KV 130 and mA 25. Multiplanar image reconstruction of the right hind limb was obtained using soft tissue and bone window reconstruction at a slice thickness of 0.6 mm.

The scans were performed on the day of implantation of the materials (day 0), and 14 days, 28 days, 56 days, and 90 days after implantation using the same protocol.

### 2.7. Histopathological Analysis of the Bones

Tissue specimens of the femur were collected at 14, 28, 56, and 90 days, and then preserved in 10% buffered neutral formaldehyde for 24 h. To protect the periosteum, the femur was extracted, and the surrounding muscles were carefully removed. This technique was used to isolate the femur for further histological examination while preserving the integrity of the periosteum and the bone. Histological sections from femoral defects were obtained directly from the site where the defects were created. Transverse planes were used to create the sections. To assess the effect of the biomaterials on the surrounding tissues and overall healing, the investigation encompassed both the primary defect area and peripheral areas. After fixation, the tissues underwent conventional histopathology processing. For examination, the paraffin-embedded samples were cut into 2-micrometer-thick slices and stained with Hematoxylin and Eosin (H&E) and Masson Trichrome (MT). Two separate pathologists evaluated the slides utilizing an Olympus BX40 microscope (Olympus Europa SE&Co, Hamburg, Germany). The photographs were captured with an Olympus SC 180 digital camera (Olympus Europa SE&Co, Hamburg, Germany) and prepared using Olympus CellSens, a specialized image acquisition and processing program.

On specific days, the samples were analyzed and contrasted. Both the C1 and C2 groups underwent a comparison with the Blank group.

## 3. Results

### 3.1. Biomaterials

A prior investigation thoroughly studied and reported on the microstructural features and the composition of the bioproducts. Furthermore, before the in vivo trials were created, early in vitro research was carried out. The research of Ardelean et al. provides all the information required [35].

After 90 days, the biomaterials remained unchanged and showed no signs of deterioration. Comparing the product to the compact bone with X H.U., the computed tomography scan revealed a greater radio-opacity. Furthermore, since the materials used possess a very high resistance to deformation or cutting, they were taken out of all samples before paraffin embedding.

### 3.2. Blood Tests

#### 3.2.1. Hematological

The complete blood counts in all groups showed normal values equivalent to those in the control group (C0), exhibiting no statistically significant differences. The hematological profile data from days 1 through 90 of the study are displayed in Table 1 and Table 2. The use of bioproducts did not negatively impact blood hematology.

#### 3.2.2. Biochemical

The biochemical profile of the whole blood was analyzed on the first and last days of this study; the results are subsequently presented in Table 3 and Table 4. The biochemical test findings showed no abnormalities of any kind, including liver or kidney impairment.

### 3.3. Surgical Procedure

A defect the size of the biomaterial was created in the cortical bone, which also impacted the medullar space. During the procedure, no complications occurred. A minor hemorrhage developed, but was maintained under control when a cold chloride solution was applied. Additionally, no signs of fracture were visible.

The entire procedure took an average of 25 min to execute, but after getting used to the method, the time was considerably shorter, saving up to 10 min.

### 3.4. Imaging

A 16-channel Siemens CT Somatom Scope was used to perform helical CT scanning of the whole body.

**Figure 4:**
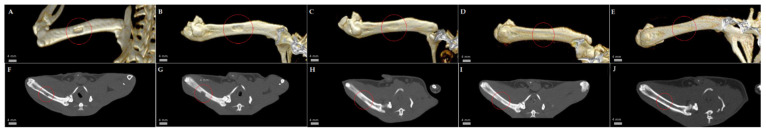
CT images showing the evolution of bone defect healing in the control rats. Three-dimensional reconstruction of the bone (**A**–**E**) and axial view (**F**–**J**): aspect from day 1 (**A**,**F**); aspect from day 14 (**B**,**G**); aspect from day 28 (**C**,**H**); aspect from day 56 (**D**,**I**); and aspect from day 90 (**E**,**J**); the area of the bone defect is marked with a red circle. Scale bar: 4 mm.

For the control rats, the bone defects were identified on the scan from day 0; it was 4.1 mm long on the bone axis, 3.1 mm deep, and 2.1 mm wide, and it involved the compact and medullary bone of the right femoral diaphysis. On the scan on day 14, the defect maintained its length and width and healing was observed from the depth, the depth being 2.8 mm; on the scan from day 28, bone healing was still evident and although the length and width of the defect remained the same, the depth of the defect decreased and was 1.6 mm; on the scan from day 56, the bone defect was no longer visible on the 3D reconstruction and from the axial compact perspective the bone was restored, the defect area showing only a slight decrease in radio-opacity; finally, at the scan on day 90, both the medullary and cortical bone were completely healed without signs of vicious callus or low radio-opacity (Figure 4).

**Figure 5:**
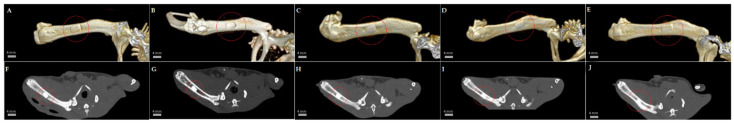
CT images showing the evolution of bone defect healing in the rats treated with the C1 material. Three-dimensional reconstruction of the bone (**A**–**E**) and axial view (**F**–**J**): aspect from day 1 (**A**,**F**); aspect from day 14 (**B**,**G**); aspect from day 28 (**C**,**H**); aspect from day 56 (**D**,**I**); and aspect from day 90 (**E**,**J**); the area of the bone defect is marked with a red circle. Scale bar: 4 mm.

For the rats in which the C1 material was implanted on day 0, the material implanted at the level of the right femoral diaphysis was identified as having a length of 4.1 mm, a thickness of 3.1 mm, and a width of 2.1 mm and showed a higher radio-opacity than the compact bone with X H.U, and the muscle reaction zone at the level of the election site; at the scan on day 14, the implanted material was in the same position and of the same size, there was a minimal soft tissue reaction, and the compact bone was not healed; on the scan from day 28, the compact bone included the tested material with the exception of the proximal edge and the soft tissue no longer showed a reaction; on the scan from day 56, the compact bone covered the tested material with the exception of the proximal edge, and no defective callus reactions or soft tissue reactions were visible at that level; on the scan from day 90 in the area of the implant, the bony compact was completely healed and no defective callus reactions or soft tissue reactions were visible at that level (Figure 5).

**Figure 6:**
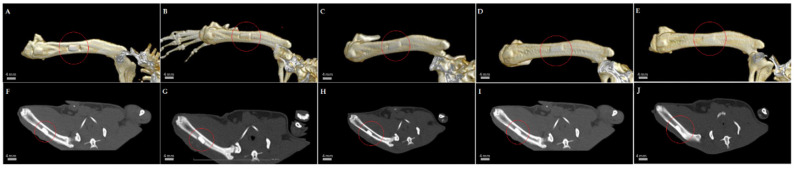
CT images showing the evolution of bone defect healing in the rats treated with the C2 material. Three-dimensional reconstruction of the bone (**A**–**E**) and axial view (**F**–**J**): aspect from day 1 (**A**,**F**); aspect from day 14 (**B**,**G**); aspect from day 28 (**C**,**H**); aspect from day 56 (**D**,**I**); aspect from day 90 (**E**,**J**); the area of the bone defect is marked with a red circle. Scale bar: 4 mm.

For the rats in which the C2 material was implanted on day 0, the material implanted at the level of the right femoral diaphysis was identified as having a length of 4.1 mm, a thickness of 3.1 mm, and a width of 2.1 mm, and showed a higher radio-opacity compared to the compact bone with X H.U and the muscle reaction area at the level of the election site; at the scan on day 14, the implanted material was in the same position and of the same size and there was a minimal soft tissue reaction, the compact bone was not healed; on the scan from day 28, the compact bone incorporated the tested material and the soft tissue no longer showed a reaction; and on the scans from days 56 and 90, the compact bone covered the tested material and no defective callus reactions or soft tissue reaction were visible at that level (Figure 6).

In conclusion, from the point of view of CT scans, in rats treated with the C2 material, healing of the bone defect took place faster (28 days) compared to rats treated with the C1 material (56 days). No abnormal reactions in either soft tissue or bone tissue were observed in any of the materials.

### 3.5. Histopathological Examination

Prior to the paraffin embedding, the materials used to close the bone defect were removed from all samples, being too hard to section. In all the analyzed preparations, a cavity indicating a lack of substance was observed, corresponding to the material used. No phenomena of absorption or incorporation of the biomaterial were identified.

On day 14, the beginning of bone regeneration was observed in the C1 individuals; trabeculae of newly formed bone, immature woven bone, and gaps with hematogenous bone marrow could be identified. The inflammatory infiltrate was absent, and there was no fibrosis. A marked difference was noted in the C2 group, where, both at the level of the bone defect and the rest of the bone cortex, there were numerous collagen fibers, increased numbers of reactive fibroblasts, along with numerous blood vessels of different calibers (granulation tissue) and a diffuse inflammatory infiltrate dominated by polymorphonuclear cells. A focus of necrosis was present, with cellular and nuclear debris, a minimal interstitial hemorrhage, and the presence of fibrin and interstitial edema. Multifocal foci of cartilaginous metaplasia were identified; the transformation of fibrous tissue into mature cartilaginous tissue and the latter into newly formed bone lamellae (immature bone) were also observed.

A similar aspect was observed at 28 days, with the same biomaterial, where a traumatic bone fracture of the femur was noted with a significant loss of bone matrix, which extended beyond the edges of the bone defect. At the fracture site, a hematoma composed of erythrocytes mixed with fibrin was observed, surrounded by proliferative mesenchymal cells (callus) containing multiple variable blood vessels, fragments of bone tissue, collagen fibers, and rare multinucleated cells (osteoclasts). The bone tissue consisted of an increased number of osteoblasts, osteocytes, and rare osteoclasts. The subperiosteal bone tissue proliferates, composed of irregular and densely packed collagen fibers, formed a focus of hyaline cartilage adjacent to the fracture. It was unclear whether the biomaterials used caused the fractures in both individuals, or if other factors, such as the bone defect or the metabolic status of the individuals, were involved. In the C1 group, immature bone was identified, which extended from the subperiosteal level to the center, without fibrosis or inflammation.

At 56 days, the aspect of the control group was that of woven bone trabeculae, composed of dense collagen bundles and numerous osteocytes, similar to that identified in the C1 group. In the C2 group, a bone callus was identified, consisting of osteochondral trabeculae, varying in size, shape, and orientation. Dense fibrous connective tissue intersected with the trabeculae toward the periosteal surface, accompanied by a small number of plasma cells, lymphocytes, and hemosiderin-laden macrophages.

On the last day of the experiment, day 90, a complete regeneration of the bone tissue was observed in the control individual, with the created bone defect being completely closed. The width of the cortical bone was not identical along the entire circumference of the bone, being reduced at the level of the defect. In both the C1 and C2 groups, the cavity corresponding to the material used was completely delimited by newly formed bone tissue, composed of dense collagen fibers and numerous osteocytes. The medullary area was reduced in size, and we were able to observe multifocal islands of osteoid detached from the newly woven bone formed. Also, a complete regeneration of the periosteum was noted, which closed the bone defect, entrapping the biomaterial used.

**Figure 7:**
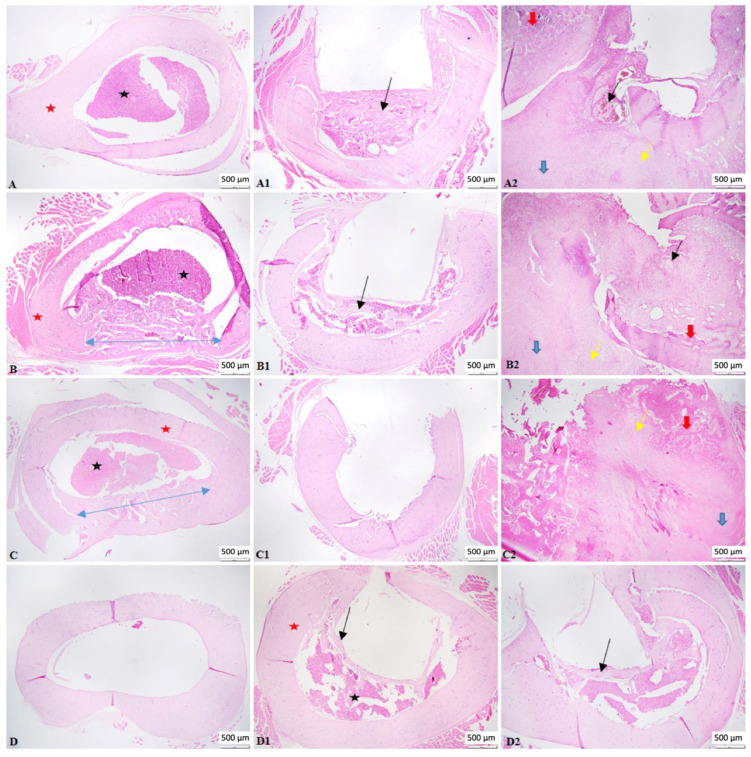
Photomicrographs of bone tissue—complete bone regeneration was observed in the control group; traumatic bone fracture was noted in all animals treated with (**C2**); by the end of the experiment, the cavity corresponding to the material used was completely delimited by newly formed bone tissue. (**A**–**D**) control group; (**A1**–**D1**) group treated with C1; (**A2**–**D2**) group treated with (**C2**). ((**A**–**C**)—blue arrow indicates bone defect); ((**A1**,**B1**,**D1**)—black arrow indicates woven bone); (black star indicates bone marrow; red star indicates compact bone); (**A2**–**C2**)—photomicrographs of callus, with blue arrows indicating collagen fibers, red arrows indicating bone trabeculae, black arrows indicating hematoma, and yellow arrows indicating cartilaginous metaplasia); (Duration: (**A**–**A2**) 14 days; (**B**–**B2**) 28 days; (**C**–**C2**) 56 days; (**D**–**D2**) 90 days). H&E, scale bar = 500 µm.

**Figure 8:**
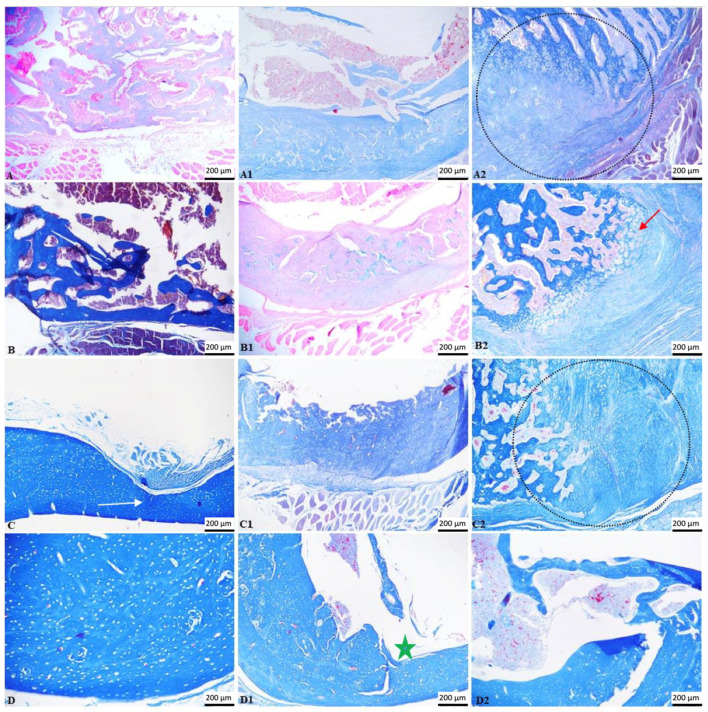
Photomicrographs of bone tissue. Bone regeneration was observed in the control group (white arrow); bone fracture and callus formation (delimited areas); cartilaginous metaplasia (red arrow) was noted in all animals treated with (**C2**); by the end of the experiment, the cavity corresponding to the material used was completely delimited by bone tissue (green star). (**A**–**D**) control group; (**A1**–**D1**) group treated with (**C1**); (**A2**–**D2**) group treated with (**C2**). Duration: (**A**–**A2**) 14 days; (**B**–**B2**) 28 days; (**C**–**C2**) 56 days; (**D**–**D2**) 90 days. TM, scale bar = 200 µm.

**Figure 9:**
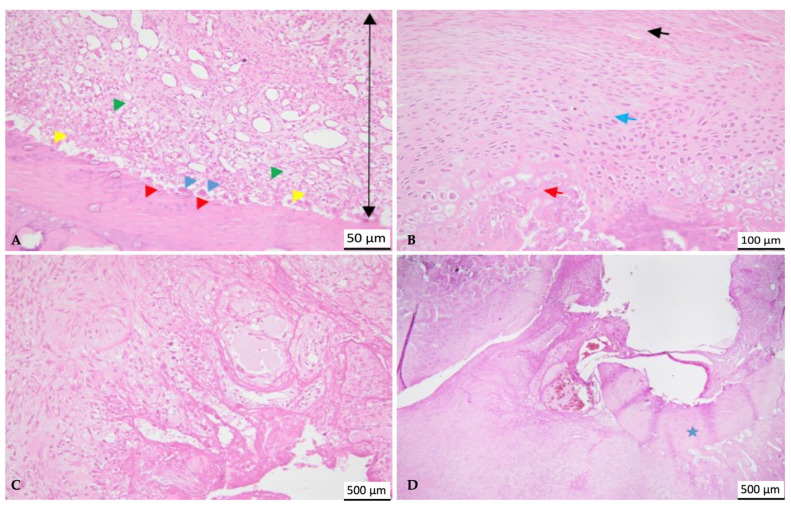
Detailed photomicrographs of bone tissue. (**A**)—granulation tissue (double arrow), containing multiple variable blood vessels and inflammatory cells (green arrowhead), accompanied by fragments of bone tissue, with rare osteoclasts (blue arrowhead), an increased number of osteoblasts (yellow arrow) and osteocytes (red arrowhead); H&E, Scale bar = 50 µm; (**B**)—endochondral ossification (fibrous tissue—black arrow; cartilaginous tissue—blue arrow; bone lamellae—red arrow); H&E, scale bar = 100 µm; (**C**)—hematoma with interstitial edema, fibrin, and granulation tissue; H&E, scale bar = 500 µm; (**D**)—fracture callus, surrounding compact bone tissue (star); H&E, scale bar = 500 µm.

Throughout the entire experiment (Figure 7), no necrotic or inflammatory changes were observed in the bone marrow for any of the materials used (Figure 8). Also, the biomaterials did not seem to excite any inflammatory or rejection response; they were well tolerated by the mice (Figure 9).

## 4. Discussion

As potential options for more extensive bone tissue repair and support, biocompatible implants have garnered a lot of interest. Our products are a mixture of inorganic and organic components that, when combined, generate biocompatible biomaterials. This study assesses how low-molecular-weight monomers (Bis-GMA, TEGDMA, UDMA, and HEMA) interact with the filler fraction, which is composed of 65% silica, hydroxyapatite, barium glass, and fluor aluminosilicate glass. The experimental cementing materials C1 and C2 were produced by distributing the particles in the organic phase. Bis-GMA resin offers mechanical strength while UDMA increases elasticity and strength [49]. TEGDMA lowers viscosity to improve handling [50] and HEMA acts as an adhesion promoter, strengthening the adhesive’s binding to the surrounding structures [51]. Excellent toughness and radio-opacity are features of barium glass [52]. At the same time, silica improves polishability and wear resistance [53].

Composites [45,46] for dentistry have been rapidly evolving over recent years, leading to their widespread usage as cosmetic tooth-like restorations [54,55]. We should not forget that dental composites might eventually be employed for bone augmentation, even though this application has not received as much interest. Critical-size bone deformities may potentially benefit from the usage of this type of bioproduct if they have been determined safe for use and biocompatible [56]. In this study, we investigated the osteo-inductive qualities, biocompatibility, and safety of composite cements when used as scaffolds in skeletal anomalies. The use of sub-critical defects can significantly affect the ability to assess the material’s osteo-inductive capacity. Determining inductivity might be difficult in models when control defects are anticipated to heal completely over time. In this research, we can conclude that our model can reliably demonstrate that the biomaterial does not impair osteogenic functions.

We found that our bioproducts remained unchanged throughout the ninety-day investigation. Based on the products’ chemical composition and the polymerization approach, neither biodegradation nor absorption of the products was observed. In a previous study, we investigated our products without polymerization on cutaneous defects, and the skin was able to absorb the biocomponents and heal effectively. During the polymerization process, the monomers create a strongly cross-linked system that is immune to decomposition by body fluids and enzymes. Fillers possess restricted dissolution qualities, reducing physiological disintegration, and are mechanically strong while remaining chemically inert. Prior research on hydroxyapatite has demonstrated that the addition of ceramic in the polymeric matrix affects the material’s mechanical characteristics, speed of decomposition, and biological behavior in a dose-dependent manner [57,58,59].

Materials that fail to degrade properly can disrupt the natural bone healing process, potentially resulting in incomplete integration or prolonged complications. Non-degradable materials may also trigger chronic inflammation or adverse responses, ultimately jeopardizing the success of bone repair [7,8]. In this study, we examined the bone’s reaction over a 90-day period and no side effects were seen. We acknowledge that the study duration is a limitation and future research should extend the observation period to achieve more comprehensive outcomes.

As previously mentioned, our polymerization approach preserves the mechanical strength of bone cements, beyond the mechanical limit of the bone. The majority of ceramic skeletal replacements are calcium-based and consist of a combination of tricalcium phosphate (TCP) and hydroxyapatite (HAP). Thus, it appears to become more and more likely to employ these composite cements scaffolds in living skeletal tissue.

The new bone starts chemical processes right after the surgery ends. First, the bone triggers a defense mechanism that attracts immune cells, and then the host mesenchymal cells migrate to the graft area through chemotaxis. Afterward, depending on several osteo-inductive signals, stem endogenous cells develop into chondroblasts and osteoblasts. Debris disintegration and revascularization take place subsequently. Ultimately, the restoration of bone takes place [60,61]. When put into the bone, the bioproducts have been observed to exhibit osteogenic capabilities. Nevertheless, the rate of resorption and disintegration is slower than that of spontaneous bone repair.

Accelerating the biomaterial’s disintegration and enhancing the scaffold’s resorption are two potential strategies that we will take into consideration for further studies [62,63,64]. However, one of this study’s limitations is that bone healing failure may not be recognized until 15 weeks, which is three times longer than it takes for a normal fracture to heal [29]. It is important to note that the biomaterial C1 did not exhibit any fractures at the defect site, whereas the biomaterial C2 experienced fractures and elicited heightened healing responses. The cause of these fractures remains unclear, and further investigation is required to elucidate this issue. We consider the small number of rats used in the research a limitation, as a larger sample size would provide more robust data. Despite having all of these features, the products have proven to be biocompatible [65,66].

To demonstrate the biocompatibility of the composites, preliminary in vitro research on stem cells was conducted, followed by in vivo subcutaneous and intramuscular tests, before creating femoral defects. The bioproducts in both trials did not exhibit any evidence of rejection or cytotoxicity. Furthermore, in vitro tests demonstrated that the composite cements C1 and C2 display osteo-inductive behavior [35]. This ability can be evaluated through histopathological assessment.

Evaluating osteointegration is challenging because cement is highly dense, which may limit its infiltration capacity. Additionally, material removal during sectioning, although necessary due to the hardness of the cements, hinders the ability to accurately demonstrate cellular interaction. Yet, the bioproducts appear to be exhibiting the characteristics of osteoconductivity, as evidenced by the surrounding tissue’s ability to close around it and its lack of resorption or retractions.

As was previously indicated, even though the defect was created in the cortical bone, it also affected the medullar area given the size of the biomaterials. In the bone marrow, mesenchymal stem populations originate from the medullary cavity. The destruction of the medullary space may impede the development of these precursor cells and thus affecting the bone’s ability to heal [67]. None of these issues were discovered throughout our investigation. There were no indications of rejection, and the medullar space continued to generate progenitor cells.

Histopathology continues to be among the gold standards for evaluating biological compatibility for the clinical usage of bioproducts [68]. Bone segments were examined histopathologically at 14, 28, 56, and 90 days. Each specimen showed evidence of the bone defect. In all research subjects, tissue growth was evident; nevertheless, none of the biomaterials were absorbed after 90 days. During this day, the tissue allowed the biomaterial to integrate and interact. Individuals with trabeculae of newly formed bone, immature woven bone, and gaps where hematogenous bone marrow may be detected exhibited the onset of bone regeneration on day 14. On day 27, immature bone was visible, unaltered by fibrosis or inflammation and spreading from the subperiosteal level to the center. The characteristic observed at 56 days was woven bone trabeculae, which were made up of many osteocytes and thick collagen filaments. On day 90, newly generated bone tissue, consisting of many osteocytes and thick collagen fibers, totally delimited the cavity corresponding to the material employed. The size of the medullary region decreased, and multifocal osteoid islands that had separated from the freshly produced woven bone were noticed. Furthermore, the whole periosteum’s regrowth was visible, sealing the bone defect and encasing the biomaterial. It is important to note that at the beginning of day 14, the C1 biomaterial provided superior results compared to the C2 biomaterial. Nevertheless, without causing a granulomatous reaction, the biomaterials demonstrated local biocompatibility. Despite requiring a lot of space, both composites encouraged positive biological effects and did not inhibit bone formation. Our subjects exhibited either no acute inflammatory cells or low levels of inflammation. A non-existent fibrous barrier surrounding the biomaterial and spontaneous regrowth of bone in proximity of the product was observed. There were no indications of necrosis or notable negative responses, which suggested positive biocompatibility.

The fact that the control in this model exhibited complete healing makes it challenging to compare these materials beyond evaluating their biocompatibility.

Rats were employed as a biological resource in this investigation. The ages of our individuals ranged from ten to sixteen weeks. Rats were selected based on their similarities with humans in terms of skeletal structure, reconstructive methods, and healing systems [69]. Even if tissue strength reduces with age, any age-related change may be minimal [70], since rats do not acquire complete ossification until they are one year old [71]. The study was conducted on the right femur to remove any further variations. All subjects were kept under the same conditions.

To validate the material’s effectiveness and suitability for human clinical trials, future research should also assess its performance in larger animal models. All things considered, this work offers a solid platform for further studies and development of bioproducts-based treatments for bone reconstruction.

Since bone cement is a foreign component of the bone cement–prosthesis system, it is critical to comprehend the variables that might cause it to prematurely lose its mechanical qualities, which could cause the prosthesis to loosen. This feature, however, is considered a limitation of our study because it was not thoroughly examined in this research.

Some of the limitations of our study include the relatively small sample size of rats, the modest size of the bone defect, and the insufficient data on biomaterial degradation and mechanical strength. These constraints have made it difficult for us to obtain more thorough judgments about how effective bone cement is.

We intend to conduct a more thorough study to overcome these issues. Future studies will evaluate bone lesions of different sizes, include a bigger batch of rats, and collect more detailed information on mechanical characteristics and biomaterial degradation.

## 5. Conclusions

Osteocompatibility was demonstrated by both biomaterials.

The physico-chemical properties of the bioproducts fit the needs of the bone. CT scans confirmed the bioproducts’ durability and mechanical strength.

The histological analysis showed no evidence of necrosis or rejection and instead supported the osteo-inductive feature of the biomaterials.

This could potentially be the starting point of a research project investigating the application of dental biomaterials in the production of scaffolds for bone defects.

## Figures and Tables

**Table 1:** Mean and Standard Deviation of measured hematological parameters from the first day of the research. Statistics performed utilizing one-way ANOVA and two-way ANOVA [46,47].

MEAN ± S.D.				
Analyte	Blank	C1	C2	References
WBC 10^9^ cells/L	7.23 ± 0.84	7.36 ± 0.96	7.65 ± 0.73	2.10–19.50
LYM 10^9^ cells/L	4.95 ± 0.37	5.03 ± 0.24	5.00 ± 0.42	2.00–14.10
MON 10^9^ cells/L	0.41 ± 0.31	0.20 ± 0.09	0.51 ± 0.32	0.00–0.98
NEU 10^9^ cells/L	1.98 ± 0.85	2.19 ± 0.96	2.36 ± 0.80	0.10–5.40
LYM %	68.57 ± 5.26	68.85 ± 6.40	67.28 ± 2.80	0–100
MON %	4.38 ± 1.00	3.85 ± 0.79	4.30 ± 1.20	0–100
NEU %	29.52 ± 7.70	27.43 ± 8.07	34.43 ± 3.34	0–100
RBC 10^12^ cells/L	8.11 ± 1.26	7.33 ± 0.73	8.86 ± 0.83	5.30–10
HGB g/dL	15.53 ± 2.42	14.01 ± 1.32	16.93 ± 1.70	14–18
HTC %	42.06 ± 5.42	38.99 ± 3.89	45.54 ± 2.77	35–52
MCV fl	52.23 ± 1.14	52.00 ± 0.71	52.10 ± 1.33	50–62
MCH pg	20.70 ± 1.34	20.15 ± 1.33	20.35 ± 1.49	16–23
MCHC g/dL	37.22 ± 1.36	36.80 ± 0.85	37.23 ± 1.57	31–40
PLT 10^9^ cells/L	647.83 ± 25.39	659.75 ± 15.11	648.00 ± 30.63	500–1370

**Table 2:** Mean and Standard Deviation of measured hematological parameters from the last day of the research. Statistics performed utilizing one-way ANOVA and two-way ANOVA [46,47].

MEAN ± S.D.				
Analyte	Blank	C1	C2	References
WBC 10^9^ cells/L	6.88 ± 0.43	7.10 ± 0.81	7.27 ± 0.71	2.10–19.50
LYM 10^9^ cells/L	5.20 ± 1.07	5.23 ± 0.48	5.08 ± 0.94	2.00–14.10
MON 10^9^ cells/L	0.53 ± 0.32	0.29 ± 0.23	0.54 ± 0.31	0.00–0.98
NEU 10^9^ cells/L	2.97 ± 0.96	2.55 ± 1.13	2.95 ± 0.89	0.10–5.40
LYM %	74.07 ± 5.17	70.48 ± 6.09	71.28 ± 2.36	0–100
MON %	4.83 ± 0.82	4.09 ± 0.66	4.65 ± 1.14	0–100
NEU %	33.87 ± 13.22	29.93 ± 9.87	38.06 ± 3.03	0–100
RBC 10^12^ cells/L	7.21 ± 0.76	7.03 ± 0.67	8.11 ± 1.15	5.30–10
HGB g/dL	15.88 ± 1.75	14.57 ± 1.61	17.01 ± 1.23	14–18
HTC %	42.06 ± 4.38	40.61 ± 4.80	44.91 ± 3.33	35–52
MCV fl	57.48 ± 1.12	54.63 ± 2.83	54.66 ± 2.88	50–62
MCH pg	21.45 ± 2.01	20.54 ± 1.91	20.53 ± 1.83	16–23
MCHC g/dL	36.68 ± 1.26	36.68 ± 1.24	36.95 ± 1.33	31–40
PLT 10^9^ cells/L	734.17 ± 21.36	707.25 ± 15.11	702.75 ± 30.47	500–1370

**Table 3:** Mean and Standard Deviation of measured biochemical parameters from the first day of the research. Statistics performed utilizing one-way ANOVA and two-way ANOVA [46,47].

MEAN ± S.D.				
Analyte	Blank	C1	C2	References
ALB g/dL	4.34 ± 0.29	4.46 ± 0.28	4.39 ± 0.34	4.1–5.4
TP g/dL	7.54 ± 0.61	7.82 ± 0.50	7.42 ± 0.64	6.4–8.5
TB mg/dL	0.07 ± 0.11	0.08 ± 0.13	0.10 ± 0.12	0.0–0.6
ALT U/L	30.85 ± 1.77	30.50 ± 2.06	32.03 ± 0.67	26–37
ALP U/L	112.07 ± 17.39	104.83 ± 16.93	123.35 ± 8.46	70–132
CREA mg/dL	0.92 ± 0.30	0.88 ± 0.29	1.03 ± 0.27	0.5–1.4
UREA mg/dL	38.08 ± 1.80	37.74 ± 1.86	39.03 ± 1.45	34.28–40.70
GLU mg/dL	131.15 ± 7.74	132.44 ± 8.17	138.14 ± 3.62	114–143
CA mg/dL	11.29 ± 0.31	11.31 ± 0.37	11.40 ± 0.22	10.5–13
PHOS mg/dL	9.90 ± 2.64	8.98 ± 2.80	11.75 ± 0.32	5–13
K mmol/L	6.60 ± 0.58	6.53 ± 0.68	6.84 ± 0.33	5.3–7.5
NA mmol/L	147.27 ± 2.36	149.30 ± 2.10	142.10 ± 2.85	143–150

**Table 4:** Mean and Standard Deviation of measured biochemical parameters from the last day of the research. Statistics performed utilizing one-way ANOVA and two-way ANOVA [46,47].

MEAN ± S.D.				
Analyte	Blank	C1	C2	References
ALB g/dL	4.86 ± 0.42	4.98 ± 1.42	4.82 ± 0.39	4.1–5.4
TP g/dL	7.71 ± 0.48	7.50 ± 2.12	7.72 ± 0.48	6.4–8.5
TB mg/dL	0.25 ± 0.11	0.20 ± 0.13	0.16 ± 0.10	0.0–0.6
ALT U/L	34.10 ± 2.28	33.11 ± 2.45	34.03 ± 2.80	26–37
ALP U/L	111.82 ± 16.01	109.11 ± 12.76	106.16 ± 9.47	70–132
CREA mg/dL	1.25 ± 0.09	1.21 ± 0.38	1.26 ± 0.09	0.5–1.4
UREA mg/dL	38.64 ± 1.73	37.38 ± 1.93	38.76 ± 1.70	34.28–40.70
GLU mg/dL	126.37 ± 8.75	119.89 ± 8.42	115.77 ± 7.09	114–143
CA mg/dL	11.99 ± 0.42	11.94 ± 0.31	11.87 ± 0.64	10.5–13
PHOS mg/dL	9.78 ± 0.96	8.23 ± 1.93	7.62 ± 1.24	5–13
K mmol/L	6.99 ± 0.30	6.86 ± 1.55	7.06 ± 0.26	5.3–7.5
NA mmol/L	147.53 ± 1.51	148.35 ± 2.45	146.70 ± 2.19	143–150

## Data Availability

Data are contained within the article.
